# Naturally occurring and added sugar in relation to macronutrient intake and food consumption: results from a population-based study in adults

**DOI:** 10.1017/jns.2017.3

**Published:** 2017-03-08

**Authors:** Niina E. Kaartinen, Minna E. Similä, Noora Kanerva, Liisa M. Valsta, Kennet Harald, Satu Männistö

**Affiliations:** Department of Health, National Institute for Health and Welfare, PO Box 30, FI-00271 Helsinki, Finland

**Keywords:** Added sugar, Sucrose, Fructose, Food consumption, Adults, DILGOM, DIetary, Lifestyle and Genetic determinants of Obesity and Metabolic syndrome, E%, percentage energy, EI, energy intake, SSB, sugar-sweetened beverages

## Abstract

Associations between sugar intake and the remaining diet are poorly described in modern food environments. We aimed at exploring associations of high naturally occurring and added sugar intakes with sociodemographic characteristics, intake of macronutrients, fibre and selected food groups. Our data comprised 4842 Finnish adults aged 25–74 years, who participated in the population-based DIetary, Lifestyle and Genetic determinants of Obesity and Metabolic syndrome (DILGOM) study. Diet was assessed by a validated 131-item FFQ. The food item disaggregation approach was used to estimate sucrose and fructose intakes from natural sources (naturally occurring sugar) and all other sources (added sugar). Sex-specific trends in macronutrient, fibre and food group intakes across sugar type quartiles were determined with general linear modelling adjusting for age, energy intake, leisure-time physical activity, smoking, education and BMI. Overall, results were similar across sexes. Young age was found to be a determinant of higher added sugar and lower naturally occurring sugar intakes (*P* < 0·0001). High added sugar intake was associated with low fibre intake (*P* < 0·0001) accompanied with lower fruit (*P* < 0·0001 women; *P* = 0·022 men) and vegetable consumption (*P* < 0·0001) and higher wheat consumption (*P* = 0·0003 women; *P* < 0·0001 men). Opposite results were found for naturally occurring sugar. Butter consumption increased by 28–32 % (*P* < 0·0001) when shifting from the lowest to the highest added sugar intake quartile, while a decrease of 26–38 % (*P* < 0·0001) was found for naturally occurring sugar. Therefore, the associations of sugar types with dietary carbohydrate and fat quality seem opposing. Proper adjustments with dietary variables are needed when studying independent relationships between sugar and health.

The significance of dietary sugars as constituents of human diets is under debate. Epidemiological studies and trials have linked high intake of sugar-sweetened beverages (SSB), one major source of dietary sugar in many countries, with poor dental health, weight gain, the metabolic syndrome, type 2 diabetes mellitus and heart disease^(^^1^^–^^4^^)^. This has contributed to the raised profile of sugar restriction on the public health agenda. In 2015, the WHO launched a strong recommendation to maintain the population average intake of free sugar under 10 % of energy (10 E%) and a conditional recommendation not to exceed 5 E%^(^^5^^)^. In the Nordic countries added sugar is recommended to stay below 10 E%^(^^6^^)^.

When considering the health effects of dietary sugars, these are typically conceptualised as free or added sugars. By definition, both of these terms include all mono- and disaccharides that are added to foods by the manufacturer, cook or consumer and sugars naturally present in honey and syrups. Free sugars also include fruit juice and fruit juice concentrates^(^^5^^,^^7^^)^. These definitions do not include intrinsic or naturally occurring sugars that are, for example, within cell walls of intact fruits, vegetables and berries. In this article we use the terms ‘added sugar’ and ‘naturally occurring sugar’ when referring to the different sugar types unless otherwise stated.

It is a general concern that high (added) sugar intakes are associated with poor-quality diets. The micronutrient dilution hypothesis represents one of the most intensively studied approaches to address the effect of high sugar intake on diet quality. Several reviews have summarised that high added sugar intakes are not consistently associated with lower intakes of micronutrients^(^^8^^)^. This has raised the need to better characterise added sugars within the total dietary context. However, studies comprehensively reporting associations between added sugar and macronutrient intake or food consumption are scarce.

Observational studies have shown strong and consistent inverse associations between total sugars (expressed as E%) and fat intake, but the association is less clear for added or naturally occurring sugar^(^^9^^)^. Overall, few studies in adults have assessed the relationship between added or naturally occurring sugar and protein, alcohol or fibre. In a cross-sectional study among 11 626 Scottish adults extrinsic sugars were inversely associated with fibre intake, and intrinsic sugar positively with intakes of protein and fibre^(^^10^^,^^11^^)^. Comparable results were obtained in two Australian studies published in 1992 and 2003 utilising comprehensive food frequency and 24-h dietary recall data, respectively^(^^12^^,^^13^^)^. These studies also revealed that high added sugar consumers were likely to have a different intake pattern of many foods such as lower intakes of wholemeal bread and fruit, higher intake of non-alcoholic beverages, but meat and poultry consumption not differing according to added sugar intake level. In a study concerning 14 709 Americans, daily servings of meat, poultry and fish decreased with increasing added sugar level (E%)^(^^14^^)^. Therefore, high added sugar intake may compromise dietary quality, which is not necessarily shared by naturally occurring sugar. Recent studies on this topic are lacking, especially studies from Northern Europe.

Given the complex nature of the modern food supply, and in order to better plan studies on sugar and health outcomes, increased understanding of the relationship between added sugars, but also naturally occurring sugar, and the overall food consumption profile is needed. This information is central in refining sugar-related communication in society.

Our objective was to construct two distinct sugar exposures on the basis of their food sources: sucrose and fructose from natural sources (fruits, berries, 100 % fruit juice and vegetables; naturally occurring sugar) and added sugar sources (all other sources; added sugar). We aimed at exploring potential associations between sociodemographic and lifestyle characteristics and sugar intake in Finnish adults. Furthermore, we investigated the cross-sectional relationship of these two sugar exposures with macronutrient and fibre intakes, as well as food consumption.

## Subjects and methods

### Participants of the DIetary, Lifestyle and Genetic determinants of Obesity and Metabolic syndrome study

The DIetary, Lifestyle and Genetic determinants of Obesity and Metabolic syndrome (DILGOM) study was conducted in the framework of the National FINRISK study, which is a Finnish population-based chronic disease risk factor monitoring survey carried out at 5-year intervals since 1972^(^^15^^)^. In 2007, a random sample of 10 000 people aged 25–74 years from the population register was drawn stratifying according to geographical area, sex and 10-year age groups. The subjects received an invitation to a health examination and a self-administered health questionnaire via mail. A total of 6258 subjects (participation rate: 63 %) participated and returned the questionnaire. All FINRISK 2007 participants were invited to the DILGOM study^(^^16^^)^. Of the invited, 5024 subjects (participation rate: 84 %) participated in the DILGOM study visit in April–June 2007. During the study visit subjects completed a FFQ and other questionnaires on health-related behaviour. Furthermore, the subjects underwent a detailed health examination.

### Diet

Food consumption was measured using a comprehensive 131-item semi-quantitative FFQ inquiring into the average use frequencies of central food groups during the previous 12 months^(^^17^^)^. Based on the National FINDIET 2007 Survey data^(^^18^^)^, each FFQ item was aggregated from 1–8 most commonly consumed foods (encoded in the food composition database) and the portion sizes were fixed sex-specifically. On the form, the portion size was specified in natural units (e.g. glass, slice) whenever possible. There were nine frequency categories for all of the items ranging from never or seldom to more than six times per d. The FFQ has been validated against 6-d food records in 510 DILGOM study subjects^(^^17^^)^. Between-method correlations for carbohydrate fractions used in the present analysis ranged from 0·39 (fructose, women) to 0·67 (dietary fibre, men). Using the same validation data and methodology^(^^17^^)^ between-method correlations for energy-adjusted fat were 0·45 and 0·38 and for protein 0·36 and 0·42 for men and women, respectively. For food groups used in the present analysis, the crude correlations ranged in men from 0·33 (meat and meat products) to 0·69 (milk and milk products) and in women from 0·30 (sugar-sweetened juice) to 0·75 (alcoholic beverages).

### The food composition database and calculation of sugar intake

The average daily consumption of foods (g/d) and the intake of energy (kJ/d) and nutrients (g/d) were calculated using in-house software and the Finnish National Food Composition Database (Fineli^®^), which is in part publicly available on the Internet^(^^18^^)^. The database is continuously updated, and includes both basic ingredients and composite foods (foods prepared at home or processed by the food industry and catering services) with individual recipes. Analysed values of nutrients are mainly based on Finnish studies. Data from the food industry and international, mainly Nordic, tables have also been used.

**Fig. 1.**
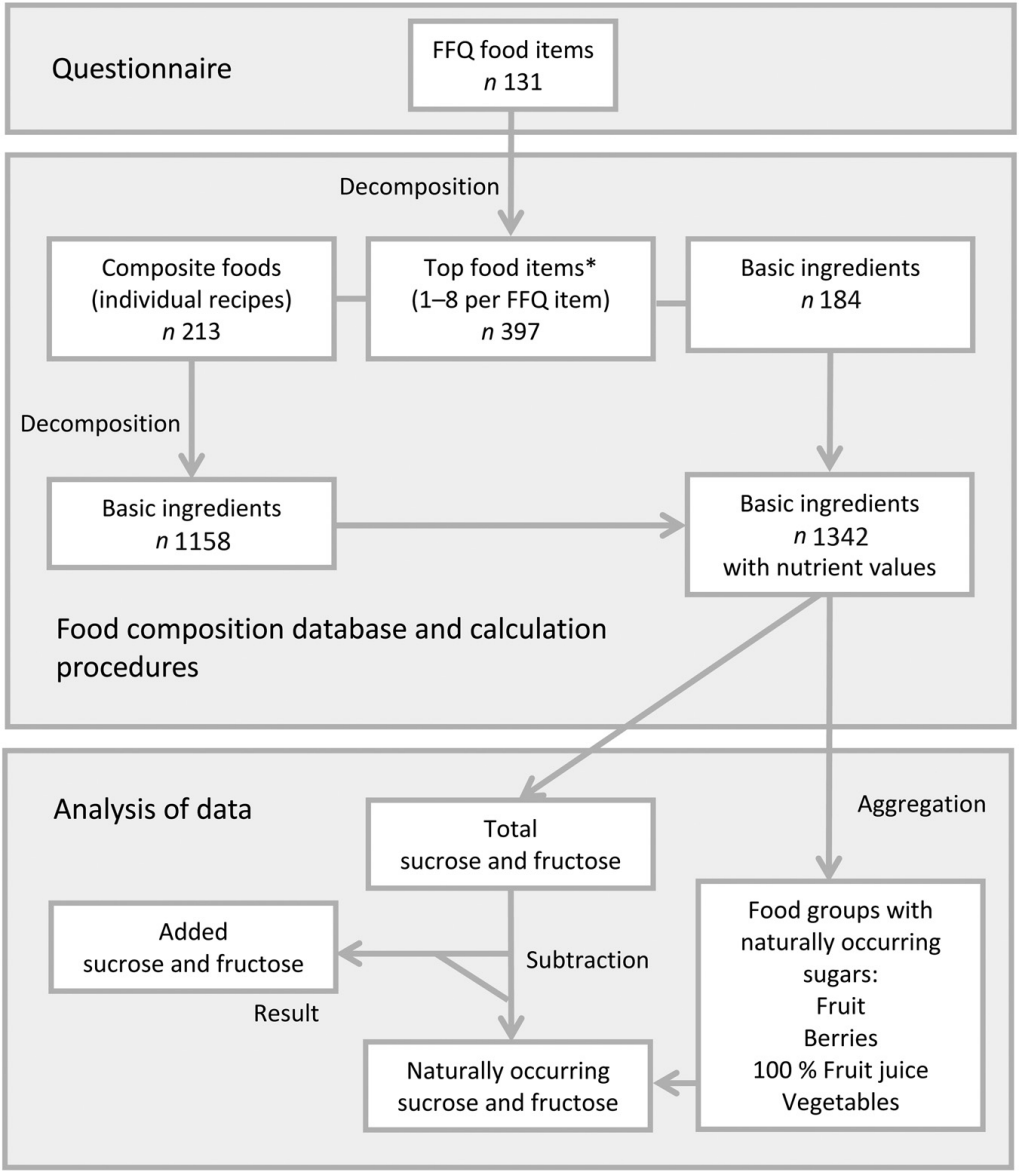
Decomposition of FFQ food items to basic ingredients using the Finnish national food composition database (Fineli^®^)^(^^18^^)^ and aggregation of basic ingredients in order to calculate naturally occurring and added sugars. * Most consumed foods per FFQ food item based on the National FINDIET 2007 Survey^(18)^.

Due to the lack of added sugar values in the database, a food disaggregation procedure was applied to yield nutrient values at the basic ingredient level (Fig. 1). For example, according to the database recipe and predefined retention factors, sweet apple tart was disaggregated to wheat flour, butter, apples and table sugar. These foods represent the ingredient-level groups ‘wheat’, ‘butter and butter mixtures’, ‘fruits’ and ‘sugar and syrups’. Using this procedure, all sucrose from the ingredient groups ‘fruits’, ‘berries’, ‘100 % fruit juices’ and ‘vegetables’ were summed up to yield the naturally occurring sucrose variable. The added sucrose variable was formed by subtracting the naturally occurring sucrose from total sucrose. Overall, the same procedures were applied for fructose.

For the analysis of associations between naturally occurring and added sugar intakes and food consumption we selected food groups illustrating both added sugar sources (sugars and syrups, sweet bakery products, sweets and chocolate, and SSB) and naturally occurring sugar sources (fruits, berries, 100 % fruit juices and vegetables). To attain an all-round view of the diets, we also included food groups illustrating the intake of fibre (rye *v.* wheat), starch (potatoes and potato products), protein and fat (meat, fish, milk products, butter and vegetable margarine) and stimulants (coffee, tea and alcoholic beverages).

### Sociodemographic and lifestyle variables and anthropometric measures

During the health examinations, trained research staff measured height (cm), weight (kg) and weight circumference (cm) according to standardised protocols^(^^19^^)^. BMI (kg/m^2^) was computed as weight (kg) divided by squared height (m). Questionnaires inquired about the subjects' education, leisure-time physical activity and smoking status. To adjust for the increase of average school years and the extension of the basic education system over time, total years of education were used to classify subjects into three educational levels (low, middle and high) according to birth year. Leisure-time physical activity was computed as a categorical variable with three levels: inactive (mainly light activities, e.g. reading, watching television), moderately active (e.g. walking, cycling or gardening at least 4 h per week) or active (physically demanding activities, e.g. running, cross-country skiing or swimming at least 3 h per week). Smoking status was defined by using three categories: never smoker, quit, and current smoker.

### The analytical sample

After exclusion of subjects with a missing FFQ (*n* 28), a totally or partly empty FFQ (*n* 74), extreme high or low energy intake (EI) corresponding to 0·5 % at both ends of the energy-intake distribution in each sex (*n* 48), missing BMI (*n* 5) and pregnant women (*n* 27), the current study yielded a sample size of 4842 subjects. Due to sex-specific FFQ portion sizes, differences in the sugar intakes, and supposedly different general heath behaviour between the sexes, all analyses were performed separately for women (*n* 2599) and men (*n* 2243).

### Statistical analyses

All analyses were performed using the SAS statistical software package version 9.3 (SAS Institute Inc.). Nutrient intakes were log (natural) transformed in order to satisfy the normality assumption and subsequently adjusted for total EI using the residual method^(^^20^^)^. The exposure variables (naturally occurring sugar, added sugar) were divided into quartiles using sex-specific cut-offs. Demographic background data were calculated both in the whole population and by quartiles of naturally occurring and added sugar intake. To take possible misreporting of EI into account, we calculated the ratio of reported EI and predicted BMR and classified subjects as either under-reporters (EI:BMR ≤ 1·14) or plausible reporters (EI:BMR > 1·14)^(^^21^^,^^22^^)^. Nutrient intakes were calculated by quartiles of sugar intakes. The *P* values for trend across quartiles were determined with general linear modelling for continuous background variables and nutrient intakes, and the *χ*^2^ test for binary background variables. For the trend analyses, subjects were assigned the median value of their sugar intake quartile, which was used as a continuous independent variable in the modelling. We applied two models in the linear trend analysis of nutrient intakes across sugar intake quartiles: age and EI (model 1); age, EI, leisure-time physical activity, smoking status, education and BMI (model 2). Model 2 was repeated without energy under-reporters.

The continuous food group variables were transformed according to the natural logarithm log(*x* + 1) to fulfil the model assumptions. The arithmetic means and 95 % CI of the transformed variables by sugar intake quartiles were calculated and antilogarithms of these were taken to yield geometric means and 95 % CI for reporting. *P* values for trend were determined with linear regression using the median values of sugar intake quartiles as continuous independent variables and each food group as continuous dependent variable at a time. We used two different sets of adjustments: age and EI (model 1); age, EI, leisure-time physical activity, smoking status, education and BMI (model 2), which was repeated without energy under-reporters.

## Results

### Background and other characteristics of subjects

Basic subject characteristics are given in Table 1. The average relative intake of total sugar (fructose and sucrose) was higher in women compared with men. On average, 43 % of women's sucrose and fructose was naturally occurring, whereas the corresponding proportion for men was 38 %. In both sexes, sucrose covered on average 45 % of the naturally occurring sugar and 91 % of the added sugar (Table 1).

**Table 1.** Characteristics and nutrient intakes of subjects in the DIetary, Lifestyle and Genetic determinants of Obesity and Metabolic Syndrome study (Medians, mean values or percentages, and 95 % confidence intervals)

	Women (*n* 2599)			Men (*n* 2243)		
	Median	Mean or %	95 % CI	Median	Mean or %	95 % CI
Characteristic						
Age (years)	53·0	52·0	51·4, 52·5	55·0	53·2	52·6, 53·7
Physically inactive subjects (%)		18·9	17·4, 20·4		18·5	16·9, 20·1
Low educated subjects (%)		31·5	29·7, 33·3		27·3	25·4, 29·1
Current smokers (%)		14·6	13·2, 16·0		20·5	18·9, 22·2
Energy under-reporters (%)		19·4	17·9, 21·0		22·6	20·8, 24·3
BMI (kg/m^2^)	25·7	26·8	26·6, 27·0	26·6	27·2	27·0, 27·3
Waist circumference (cm)	84·5	86·8	86·3, 87·3	95·5	96·5	96·0, 97·0
Nutrient intake (g/d)*						
Carbohydrate	284	284	282, 285	270	271	270, 273
Total sugar (fructose and sucrose)	83·1	85·1	84·1, 86·1	69·9	72·6	71·5, 73·7
Naturally occurring sugar†	32·7	36·4	35·6, 37·1	24·2	27·8	27·1, 28·6
Naturally occurring fructose	18·1	20·0	19·6, 20·5	13·2	15·3	14·9, 15·7
Naturally occurring sucrose	14·5	16·3	16·0, 16·7	10·6	12·5	12·2, 12·9
Added sugar‡	45·7	48·8	48·0, 49·6	42·0	44·5	43·7, 45·4
Added fructose	3·9	4·4	4·3, 4·5	3·6	4·0	4·0, 4·1
Added sucrose	41·5	44·5	43·7, 45·2	38·1	40·4	39·7, 41·2
Dietary fibre	31·3	32·1	31·7, 32·4	26·2	26·7	26·4, 27·1
Protein	102	103	102, 103	103	103	103, 104
Fat	81·5	81·9	81·4, 82·4	84·1	83·9	83·4, 84·5
Alcohol	2·6	5·2	4·9, 5·5	6·1	9·7	9·2, 10·3
Carbohydrate (E%)	50·3	50·2	50·0, 50·5	47·5	47·7	47·4, 47·9
Total sugar (E%)	14·3	14·6	14·4, 14·8	12·1	12·6	12·4, 12·8
Naturally occurring sugar (E%)	5·7	6·3	6·2, 6·4	4·1	4·7	4·6, 4·9
Added sugar (E%)	7·8	8·3	8·1, 8·4	7·3	7·8	7·7, 8·0
Dietary fibre (g/1000 kcal (4184 kJ))	13·4	13·8	13·7, 14·0	10·8	11·1	11·0, 11·3
Protein (E%)	17·7	17·8	17·7, 17·9	17·6	17·7	17·6, 17·8
Fat (E%)	30·4	30·6	30·4, 30·7	31·7	31·7	31·5, 31·9
Alcohol (ethanol) (E%)	0·7	1·4	1·3, 1·5	1·9	2·9	2·8, 3·1

E%, percentage energy.

* Nutrient intake was adjusted for total energy intake using the residual method^(^^20^^)^.

† Naturally occurring sugar is defined as all sucrose and fructose naturally present in fruits, berries, vegetables and 100 % fruit juice used as such or as a food ingredient.

‡ Added sugar is defined as sucrose and fructose originating from all other foods and food ingredient sources as the above mentioned, and was calculated as (total sucrose + fructose) – (naturally occurring sugar).

The median naturally occurring sugar intakes across quartiles ranged from 2·9 to 10·2 E% in women and from 1·9 to 8·0 E% in men. Corresponding ranges for added sugar were from 4·7 to 12·1 E% in women and from 4·1 to 11·8 E% in men (data not shown). In women, the increasing relative intake of naturally occurring sugar was associated with increasing age and decreasing BMI and waist circumference, whereas the proportions of physically inactive, low educated, currently smoking and energy under-reporting women appeared lower with increasing intakes (Table 2). Similar results were found in men, except that the association between increasing intake of naturally occurring sugar was not associated with BMI and waist circumference. In both sexes, the increasing relative intake of added sugar was associated with decreasing age, waist circumference and BMI (men only). No statistically significant association was found between increasing intake of added sugar and the proportions of physically inactive and low educated subjects in either sex. A significantly lower proportion of current smokers appeared with increasing relative intake of added sugar in men, but not women.

**Table 2.** Sociodemographic and lifestyle characteristics by lowest and highest sugar intake quartile (Q) in the DIetary, Lifestyle and Genetic determinants of Obesity and Metabolic syndrome study (Mean values or percentages, and 95 % confidence intervals)

	Naturally occurring sucrose and fructose intake quartile*					Added sucrose and fructose intake quartile†				
	Q1 (lowest)		Q4 (highest)			Q1 (lowest)		Q4 (highest)		
Characteristic	Mean or %	95 % CI	Mean or %	95 % CI	*P*‡	Mean or %	95 % CI	Mean or %	95 % CI	*P*‡
Women (*n* 2599)										
Age (years)	50·9	49·8, 52·0	53·8	52·9, 54·8	<0·0001	55·1	54·2, 56·0	50·0	48·9, 51·1	<0·0001
Physically inactive subjects (%)	27·5	24·0, 30·9	14·7	12·0, 17·5	<0·0001	18·5	15·5, 21·5	21·5	18·3, 24·7	0·26
Low educated subjects (%)	37·3	33·5, 41·0	25·9	22·5, 29·3	0·0001	31·6	28·0, 35·2	35·0	31·3, 38·7	0·12
Current smokers (%)	20·2	17·1, 23·3	13·4	10·8, 16·0	<0·0001	17·5	14·6, 20·5	13·4	10·8, 16·1	0·11
BMI (kg/m^2^)	27·2	26·8, 27·7	26·6	26·2, 27·0	0·038	27·2	26·8, 27·6	26·5	26·1, 27·0	0·063
Waist circumference (cm)	88·2	87·1, 89·3	86·1	85·2, 87·1	0·011	88·4	87·4, 89·4	85·8	84·7, 86·8	0·003
Energy under-reporters (%)§	23·6	20·3, 26·8	18·6	15·6, 21·6	0·001	22·8	19·6, 26·0	19·2	16·2, 22·3	0·022
Men (*n* 2243)										
Age (years)	52·6	51·5, 53·7	54·3	53·2, 55·5	0·007	54·5	53·4, 55·6	52·2	51·1, 53·3	0·003
Physically inactive subjects (%)	24·9	21·3, 28·5	14·0	11·1, 16·8	<0·0001	21·8	18·4, 25·2	17·1	13·9, 20·2	0·14
Low educated subjects (%)	38·1	34·1, 42·1	20·8	17·4, 24·2	<0·0001	25·0	21·4, 28·6	30·6	26·8, 34·5	0·11
Current smokers (%)	29·4	25·6, 33·2	15·9	12·9, 19·0	<0·0001	24·3	20·8, 27·9	18·1	14·9, 21·3	0·041
BMI (kg/m^2^)	27·7	27·3, 28·1	27·4	27·1, 27·8	0·59	27·8	27·4, 28·1	26·4	26·1, 26·7	<0·0001
Waist circumference (cm)	98·2	97·1, 99·3	97·1	96·1, 98·1	0·33	98·5	97·4, 99·5	94·2	93·3, 95·1	<0·0001
Energy under-reporters (%)§	29·3	25·5, 33·1	21·6	18·2, 25·0	<0·0001	27·5	23·8, 31·2	21·2	17·8, 24·6	0·013

* Naturally occurring refers to sucrose and fructose from fruits, berries, 100 % fruit juice and vegetables. The variable is energy adjusted^(^^20^^)^ and divided into sex-specific quartiles. The median values and ranges of quartiles are: Q1, 16·6 g/d, 0·78–21·9; Q2, 27·3 g/d, 22·0–32·6; Q3, 38·7 g/d, 32·7–46·8; Q4, 58·5 g/d, 46·9–205 (women); and Q1, 11·3 g/d, 0·33–15·2; Q2, 19·7 g/d, 15·3–24·1; Q3, 29·2 g/d, 24·2–36·7; Q4, 46·7 g/d, 36·8–165 (men).

† The added sucrose and fructose variable was calculated as (total sucrose + fructose) – (naturally occurring sucrose + fructose). The variable is energy adjusted^(^^20^^)^ and divided into sex-specific quartiles. The median values and ranges of quartiles are: Q1, 27·6 g/d, 6·2–34·7; Q2, 40·1 g/d, 34·7–45·7; Q3, 51·8 g/d, 45·7–59·5; Q4, 70·9 g/d, 59·6–190 (women); and Q1, 23·7 g/d, 3·8–30·8; Q2, 36·2 g/d, 30·9–41·9; Q3, 47·3 g/d, 42·0–54·1; Q4, 66·4 g/d, 54·2–215 (men).

‡ *P* values for linear trend across quartiles were determined with generalised linear modelling for continuous background variables and the *χ*^2^ test for binary background variables. The quartile median values of sucrose + fructose intakes were used as continuous independent variables in the linear modelling.

§ Energy under-reporters were defined based on the Goldberg cut-off value (≤1·14) for the ratio of reported energy intake:predicted BMR^(^^21^^,^^22^^)^.

### Macronutrients and fibre

In both sexes, the increasing relative intakes of both naturally occurring and added sugar were associated with higher intakes of carbohydrate and total sugar (sucrose and fructose), and lower intakes of protein, fat and alcohol (*P* values for trend <0·0001, Table 3, model 2). The relative intake of added sugar decreased significantly with increasing naturally occurring sugar intakes (*P* < 0·0001 in women and *P* = 0·033 in men), and the relative intake of naturally occurring sugar decreased with increasing added sugar intakes (*P* < 0·0001 in women and *P* = 0·011 in men). In both sexes dietary fibre intake increased with increasing intakes of naturally occurring sugar, while the opposite was found with increasing intakes of added sugar (*P* < 0·0001). In general, the addition of leisure-time physical activity, smoking status, education and BMI, into the model (model 2) did not affect the linear trends in nutrient intakes across sugar quartiles. The exclusion of energy under-reporters produced similar results.

**Table 3.** Nutrient intakes by lowest and highest sugar intake quartile (Q) in the DIetary, Lifestyle and Genetic determinants of Obesity and Metabolic syndrome study (Mean values and 95 % confidence intervals)

	Naturally occurring sucrose and fructose intake quartile*						Added sucrose and fructose intake quartile†					
	Q1 (lowest)		Q4 (highest)				Q1 (lowest)		Q4 (highest)			
Nutrient intake (g/d)‡	Mean	95 % CI	Mean	95 % CI	*P*§‖	*P*§¶	Mean	95 % CI	Mean	95 % CI	*P*§‖	*P*§¶
Women (*n* 2599)												
Energy (MJ/d)	9·07	8·82, 9·31	9·75	9·51, 10·00	0·0002	<0·0001	9·30	9·05, 9·55	9·56	9·32, 9·81	0·06	0·11
Carbohydrate	270	267, 272	302	299, 304	<0·0001	<0·0001	270	267, 272	300	297, 302	<0·0001	<0·0001
Total sucrose and fructose	68·4	66·7, 70·0	108	106, 110	<0·0001	<0·0001	67·3	65·6, 68·9	109	107, 110	<0·0001	<0·0001
Added sucrose and fructose	53·1	51·6, 54·7	44·4	42·8, 45·9	<0·0001	<0·0001	26·6	25·8, 27·3	76·5	75·8, 77·2	<0·0001	<0·0001
Naturally occurring sucrose and fructose	15·8	15·1, 16·5	63·2	62·5, 63·9	<0·0001	<0·0001	40·4	38·9, 41·9	32·7	31·2, 34·1	<0·0001	<0·0001
Dietary fibre	27·8	27·1, 28·4	36·6	36·0, 37·3	<0·0001	<0·0001	35·5	34·8, 36·1	28·8	28·1, 29·4	<0·0001	<0·0001
Protein	105	103, 106	99·1	97·9, 100	<0·0001	<0·0001	111	110, 112	93·4	92·4, 94·4	<0·0001	<0·0001
Fat	87·6	86·6, 88·5	74·9	73·9, 75·8	<0·0001	<0·0001	83·7	82·7, 84·7	79·6	78·6, 80·6	<0·0001	<0·0001
Alcohol	6·0	5·4, 6·6	4·9	4·3, 5·5	0·09	0·027	6·8	6·2, 7·4	3·8	3·2, 4·4	<0·0001	<0·0001
Men (*n* 2243)												
Energy (MJ/d)	11·22	10·89, 11·56	11·81	11·48, 12·15	0·047	0·05	11·36	11·02, 11·69	11·67	11·33, 12·00	0·22	0·17
Carbohydrate	257	254, 260	290	287, 293	<0·0001	<0·0001	255	252, 257	290	288, 293	<0·0001	<0·0001
Total sucrose and fructose	56·5	54·7, 58·3	93·7	91·9, 95·5	<0·0001	<0·0001	51·2	49·6, 52·8	98·0	96·4, 99·6	<0·0001	<0·0001
Added sucrose and fructose	45·5	43·8, 47·2	42·7	41·0, 44·4	0·017	0·033	22·6	21·8, 23·4	71·6	70·8, 72·4	<0·0001	<0·0001
Naturally occurring sucrose and fructose	10·6	10·0, 11·3	51·2	50·5, 51·9	<0·0001	<0·0001	28·6	27·2, 30·0	25·8	24·4, 27·2	0·009	0·011
Dietary fibre	23·7	23·1, 24·3	30·0	29·4, 30·6	<0·0001	<0·0001	28·1	27·5, 28·7	24·9	24·3, 25·6	<0·0001	<0·0001
Protein	105	104, 106	99·7	98·5, 101	<0·0001	<0·0001	110	109, 112	94·5	93·4, 95·6	<0·0001	<0·0001
Fat	88·6	87·6, 89·7	77·9	76·8, 78·9	<0·0001	<0·0001	85·4	84·4, 86·5	80·8	79·7, 81·9	<0·0001	<0·0001
Alcohol	11·5	10·5, 12·5	8·0	6·9, 9·0	<0·0001	<0·0001	13·3	12·2, 14·3	7·4	6·4, 8·4	<0·0001	<0·0001

* Natural refers to sucrose and fructose from fruits, berries, fruit juice and vegetables. The variable is energy adjusted using the residual method^(^^20^^)^ and divided into sex-specific quartiles. The median values and ranges of quartiles are: Q1, 16·6 g/d, 0·78–21·9; Q2, 27·3 g/d, 22·0–32·6; Q3, 38·7 g/d, 32·7–46·8; Q4, 58·5 g/d, 46·9–205 (women); and Q1, 11·3 g/d, 0·33–15·2; Q2, 19·7 g/d, 15·3–24·1; Q3, 29·2 g/d, 24·2–36·7; Q4, 46·7 g/d, 36·8–165 (men).

† The added sucrose and fructose variable was calculated as (total sucrose + fructose) – (naturally occurring sucrose + fructose). The variable is energy adjusted using the residual method^(^^20^^)^ and divided into sex-specific quartiles. The median values and ranges of quartiles are: Q1, 27·6 g/d, 6·2–34·7; Q2, 40·1 g/d, 34·7–45·7; Q3, 51·8 g/d, 45·7–59·5; Q4, 70·9 g/d, 59·6–190 (women); and Q1, 23·7 g/d, 3·8–30·8; Q2, 36·2 g/d, 30·9–41·9; Q3, 47·3 g/d, 42·0–54·1; Q4, 66·4 g/d, 54·2–215 (men).

‡ Values shown in the table are adjusted for age and total energy intake, except energy intake values are only age adjusted.

§ *P* values for linear trends were determined with generalised linear modelling using the median values of sugar intake quartiles as continuous independent variables.

‖ Adjusted for age and energy intake. When energy was used as a dependent variable the analyses were adjusted for age only.

¶ Adjusted for age, energy intake, leisure-time physical activity, smoking status, education and BMI.

### Food groups

In general, the associations between the two sugar types and food consumption were similar in the two models and across sexes (Tables 4 and 5). In model 2, increasing naturally occurring sugar intake was statistically significantly associated with higher intakes of fruits, berries, 100 % fruit juices and vegetables (*P* < 0·0001, naturally occurring sugar sources), whereas the consumption of added sugar sources such as sugars and syrups, sweet bakery products, sweets and chocolate (*P* = 0·0007 for women and NS for men), and SSB (women only, *P* < 0·0001) was lower. Compared with these results, opposite associations were found between increasing added sugar intake and the food sources of the different sugars. From the naturally occurring sugar sources only berries associated positively with rising added sugar intake (*P* = <0·0001), and 100 % fruit juices were not significantly associated with added sugar intake in either sex.

**Table 4.** Consumption of food ingredients (geometric means) by highest and lowest sugar intake quartiles (Q) in 2599 women participating in the DIetary, Lifestyle and Genetic determinants of Obesity and Metabolic syndrome study (Mean values and 95 % confidence intervals)

	Naturally occurring sucrose and fructose intake quartile*						Added sucrose and fructose intake quartile†					
	Q1 (lowest)		Q4 (highest)				Q1 (lowest)		Q4 (highest)			
Food consumption (g/d)‡	Mean	95 % CI	Mean	95 % CI	*P*§‖	*P*§¶	Mean	95 % CI	Mean	95 % CI	*P*§‖	*P*§¶
Sugars and syrups	13·1	12·5, 13·7	10·7	10·1, 11·2	<0·0001	<0·0001	7·4	7·1, 7·8	18·3	17·5, 19·0	<0·0001	<0·0001
Sweet bakery products	44·9	41·7, 48·5	26·7	24·7, 28·9	<0·0001	<0·0001	22·4	20·7, 24·1	50·3	46·8, 54·2	<0·0001	<0·0001
Sweets and chocolate	11·2	10·4, 12·2	9·6	8·8, 10·5	0·005	0·0007	6·0	5·6, 6·6	16·6	15·4, 17·8	<0·0001	<0·0001
Sugar-sweetened beverages	28·1	24·3, 32·6	16·9	14·6, 19·7	<0·0001	<0·0001	6·8	5·8, 7·9	71·8	62·9, 81·8	<0·0001	<0·0001
Fruits	60·2	57·2, 63·3	398	379, 419	<0·0001	<0·0001	193	180, 208	141	131, 151	<0·0001	<0·0001
Berries	25·9	24·3, 27·7	34·7	32·5, 37·0	<0·0001	<0·0001	27·5	25·7, 29·4	34·0	31·9, 36·4	<0·0001	0·0002
Fruit juices (100 %)	9·7	8·4, 11·3	83·5	73·1, 95·4	<0·0001	<0·0001	26·1	22·5, 30·3	26·7	23·0, 30·9	0·88	0·58
Vegetables	214	205, 223	341	327, 355	<0·0001	<0·0001	339	325, 354	234	224, 244	<0·0001	<0·0001
Rye	51·6	48·5, 54·9	45·4	42·7, 48·3	0·001	<0·0001	59·2	55·6, 62·9	41·3	38·9, 44·0	<0·0001	<0·0001
Wheat	65·1	62·9, 67·5	47·6	45·9, 49·3	<0·0001	<0·0001	52·1	50·2, 54·0	59·0	56·9, 61·1	<0·0001	0·0003
Potatoes and potato products	110	106, 115	91·4	87·6, 95·4	<0·0001	<0·0001	108	103, 113	92·7	88·8, 96·8	<0·0001	<0·0001
Meat and meat products	140	134, 147	118	113, 124	<0·0001	<0·0001	144	137, 150	111	106, 116	<0·0001	<0·0001
Fish, fish products and shellfish	31·6	29·7, 33·6	34·1	32·1, 36·3	0·17	0·31	39·8	37·4, 42·3	28·4	26·7, 30·2	<0·0001	<0·0001
Milk and milk products	525	502, 548	433	415, 453	<0·0001	<0·0001	450	431, 470	503	481, 525	0·001	0·004
Butter (including butter oil mixtures)	6·0	5·7, 6·3	3·7	3·5, 4·0	<0·0001	<0·0001	4·1	3·9, 4·4	5·4	5·1, 5·8	<0·0001	<0·0001
Vegetable margarine	7·8	7·2, 8·5	6·9	6·3, 7·5	0·029	0·009	8·3	7·6, 9·0	6·8	6·2, 7·4	0·0003	0·0004
Coffee and tea	445	414, 479	433	402, 466	0·72	0·35	446	414, 479	405	376, 435	0·08	0·22
Alcoholic beverages	21·6	18·5, 25·1	21·6	18·5, 25·1	0·86	0·50	31·0	26·7, 36·1	15·0	12·9, 17·5	<0·0001	<0·0001

* Natural refers to sucrose and fructose from fruits, berries, fruit juice and vegetables. The variable is energy adjusted using the residual method^(^^20^^)^. The median naturally occurring sugar intakes and ranges of quartiles are: Q1, 16·6 g/d, 0·78–21·9; Q2, 27·3 g/d, 22·0–32·6; Q3, 38·7 g/d, 32·7–46·8; Q4, 58·5 g/d, 46·9–205.

† The added sucrose and fructose variable was calculated as (total sucrose + fructose) – (naturally occurring sucrose + fructose) and energy adjusted using the residual method. The median added sugar intakes and quartile ranges are: Q1, 27·6 g/d, 6·2–34·7; Q2, 40·1 g/d, 34·7–45·7; Q3, 51·8 g/d, 45·7–59·5; Q4, 70·9 g/d, 59·6–190.

‡ Values in the table are adjusted for age and energy intake.

§ *P* values for linear trend were determined with generalised linear modelling treating the median values of sugar intake quartiles as continuous variables and each log-transformed food group as a continuous dependent variable at a time.

‖ Adjusted for age and energy intake.

¶ Adjusted for age, energy intake, leisure-time physical activity, smoking status, education and BMI.

**Table 5.** Consumption of food ingredients (geometric means) by highest and lowest sugar intake quartiles (Q) in 2243 men participating in the DIetary, Lifestyle and Genetic determinants of Obesity and Metabolic syndrome study (Mean values and 95 % confidence intervals)

	Naturally occurring sucrose and fructose intake quartile*						Added sucrose and fructose intake quartile†					
	Q1 (lowest)		Q4 (highest)				Q1 (lowest)		Q4 (highest)			
Food consumption (g/d)‡	Mean	95 % CI	Mean	95 % CI	*P*§‖	*P*§¶	Mean	95 % CI	Mean	95 % CI	*P*§‖	*P*§¶
Sugars and syrups	16·8	15·9, 17·9	14·0	13·2, 14·9	<0·0001	<0·0001	8·4	7·9, 8·8	25·4	24·2, 26·7	<0·0001	<0·0001
Sweet bakery products	51·8	47·4, 56·6	39·4	36·0, 43·0	<0·0001	<0·0001	26·7	24·5, 29·1	66·4	61·1, 72·2	<0·0001	<0·0001
Sweets and chocolate	8·2	7·5, 9·1	9·7	8·9, 10·6	0·047	0·40	5·4	4·9, 5·9	14·1	12·9, 15·3	<0·0001	<0·0001
Sugar-sweetened beverages	33·5	28·7, 39·0	40·4	34·7, 47·0	0·21	0·17	12·9	11·2, 14·8	134	118, 153	<0·0001	<0·0001
Fruits	41·9	39·1, 44·9	270	253, 289	<0·0001	<0·0001	117	107, 128	104	94·7, 113	0·033	0·022
Berries	15·3	14·0, 16·6	26·2	24·1, 28·4	<0·0001	<0·0001	16·0	14·8, 17·4	25·4	23·4, 27·5	<0·0001	<0·0001
Fruit juices (100 %)	14·0	12·3, 16·0	170	151, 192	<0·0001	<0·0001	44·6	38·6, 51·4	54·7	47·4, 63·0	0·07	0·06
Vegetables	160	153, 168	269	257, 282	<0·0001	<0·0001	248	236, 260	188	179, 197	<0·0001	<0·0001
Rye	54·9	51·4, 58·7	49·7	46·5, 53·1	0·028	0·024	58·6	54·8, 62·6	46·4	43·4, 49·5	<0·0001	<0·0001
Wheat	79·9	77·1, 82·9	68·8	66·4, 71·3	<0·0001	<0·0001	67·4	65·0, 69·9	78·0	75·2, 80·8	<0·0001	<0·0001
Potatoes and potato products	165	158, 172	136	130, 142	<0·0001	<0·0001	167	160, 175	142	136, 148	<0·0001	<0·0001
Meat and meat products	204	196, 212	179	172, 185	<0·0001	<0·0001	219	211, 227	170	164, 176	<0·0001	<0·0001
Fish, fish products and shellfish	41·5	38·8, 44·4	49·1	45·9, 52·5	0·005	0·10	53·9	50·4, 57·6	40·2	37·6, 43·0	<0·0001	<0·0001
Milk and milk products	595	564, 628	478	453, 504	<0·0001	<0·0001	545	516, 575	532	504, 562	0·47	0·33
Butter (including butter oil mixtures)	7·3	6·9, 7·8	5·4	5·1, 5·8	<0·0001	<0·0001	5·4	5·1, 5·8	6·9	6·4, 7·3	<0·0001	<0·0001
Vegetable margarine	8·9	8·1, 9·8	9·0	8·2, 9·9	0·93	0·97	10·3	9·4, 11·3	8·1	7·3, 8·9	0·001	0·001
Coffee and tea	462	430, 497	422	392, 454	0·06	0·12	425	395, 457	464	432, 500	0·12	0·017
Alcoholic beverages	76·6	64·6, 90·7	62·1	52·4, 73·6	0·08	0·009	101	85·6, 120	53·3	45·0, 63·2	<0·0001	<0·0001

* Natural refers to sucrose and fructose from fruits, berries, fruit juice and vegetables. The variable is energy adjusted using the residual method^(^^20^^)^. The median naturally occurring sugar intakes and ranges of quartiles are: Q1, 11·3 g/d, 0·33–15·2; Q2, 19·7 g/d, 15·3–24·1; Q3, 29·2 g/d, 24·2–36·7; Q4, 46·7 g/d, 36·8–165.

† The added sucrose and fructose variable was calculated as (total sucrose + fructose) – (naturally occurring sucrose + fructose) and energy adjusted using the residual method. The median added sugar intakes and quartile ranges are: Q1, 23·7 g/d, 3·8–30·8; Q2, 36·2 g/d, 30·9–41·9; Q3, 47·3 g/d, 42·0–54·1; Q4, 66·4 g/d, 54·2–215.

‡ Values in the table are adjusted for age and energy intake.

§ *P* values for trend were determined with generalised linear modelling treating the median values of sugar intake quartiles as continuous variables and each log-transformed food group as a continuous dependent variable at a time.

‖ Adjusted for age and energy intake.

¶ Adjusted for age, energy intake, leisure-time physical activity, smoking status, education and BMI.

From the other food groups rye (*P* < 0·0001 for women and *P* = 0·024 for men), potatoes, meat and milk products, butter (all *P* < 0·0001 for both sexes) and vegetable margarine (*P* = 0·009 for women and NS for men) consumptions were lower for high naturally occurring sugar intakes. Wheat consumption decreased 14–27 % from the lowest to the highest naturally occurring sugar intake quartile (*P* < 0·0001 for both sexes). Fish consumption was not associated with naturally occurring sugar intake in either women or men, and alcoholic beverage consumption only among men (inverse association, *P* = 0·009). In both sexes, the consumption of rye, potatoes, meat, fish, vegetable margarine and alcoholic beverages decreased with higher added sugar intakes. Consumption of wheat increased 13–16 % from the lowest to the highest added sugar intake quartile (*P* = 0·0003 for women and *P* < 0·0001 for men). Also, the consumption of milk products (*P* = 0·004 for women and NS for men) and butter (*P* < 0·0001 for both sexes) increased with higher intakes of added sugar. Added sugar intake was associated also with higher coffee and tea consumption in men (*P* = 0·017) but not in women.

## Discussion

This cross-sectional analysis in 4842 Finnish adults investigated associations of naturally occurring sugar and added sugar with sociodemographic characteristics and overall diet approached through macronutrient intake and consumption of selected food groups. Older age was associated with higher naturally occurring sugar intakes and lower added sugar intakes. High added sugar intake was also associated with lower fibre intake and found to potentially compromise dietary fat quality. Strengths of this study include the population-based approach, and the use of a culturally well-adopted FFQ, which is found to be reasonably valid for measuring sucrose and fructose intakes in our study population^(^^17^^)^. To our knowledge, the relationship between sugar intake and different food groups in adult populations has not been comprehensively investigated in Finland or other Nordic countries before.

Overall healthier lifestyle with regard to leisure-time physical activity and smoking was associated with high naturally occurring sugar intake. These results are in line with studies showing clustering of unhealthy, but also healthy, behaviours^(^^23^^)^. Recently, a study in 9425 New Zealand adults demonstrated a two-level clustering of fruit and vegetable intake with physical activity, sedentary behaviour and SSB consumption: optimal wellbeing was higher in healthy behaviour combinations^(^^24^^)^. In our study, young age was associated with higher added sugar intakes – a phenomenon seen in other cultures as well^(^^25^^–^^27^^)^. This strengthens the basis to keep added sugar intake reduction on the public health agenda and focusing on total diet and total lifestyle to optimise population health.

With regard to macronutrients, our results conform to studies from the 1990s reporting an inverse relationship between sugar and fat intake (sugar–fat see-saw phenomenon) when using E% of extrinsic sugar or intrinsic sugar as exposures^(^^10^^,^^12^^,^^28^^,^^29^^)^. Similar to our results, high relative added/extrinsic sugar intakes have been inversely associated with protein and alcohol intakes^(^^10^^,^^13^^)^. In these studies, naturally occurring/intrinsic sugar was, if anything, positively associated with protein intake. In our study, rising added sugar intake was associated with a greater decrease in protein intake (14–16 % difference between highest and lowest quartile) compared with naturally occurring sugar (5–6 % difference between the highest and lowest quartiles). This suggests that added and naturally occurring sugars act as markers of divergent diets accompanied by different macronutrient compositions.

Current Nordic dietary recommendations emphasise the quality of dietary carbohydrate and fat over their amount^(^^6^^)^. We found a positive association between naturally occurring sugar and fibre intake, whereas the opposite was observed for added sugar. These results were independent of age, EI, leisure-time physical activity, smoking status, education and BMI. Studies from different food cultures have shown similar results. In a Scottish study utilising FFQ data from 11 626 men and women aged 25–64 years, extrinsic sugar was associated negatively and intrinsic sugar positively with fibre intake after adjusting for important confounders^(^^11^^)^. Comparable results were also obtained using 24-h recall data in 10 417 Australian adults^(^^13^^)^, and in 15 189 Americans from the National Health and Nutrition Examination Survey (NHANES) 2003–2006^(^^30^^)^. In a Dutch study, adults adhering to the dietary guideline of <10 E% for added sugar scored higher for the healthy diet index components fibre, but also fruits and vegetables^(^^26^^)^. This is also in line with our food consumption results for fruits, berries and vegetables. Overall, results for fibre are also supported by our results for wheat and rye (to some extent). The decrease in rye consumption (highest *v.* lowest quartile) seemed smaller for naturally occurring sugar (9–12 %) than for added sugar (21–30 %).

Regarding dietary fat quality, we found only one study in which non-milk extrinsic sugars were associated with lower SFA, MUFA and PUFA intakes^(^^29^^)^. However, total sugars or sucrose also seem to have an inverse association with these different fatty acids^(^^13^^,^^31^^,^^32^^)^. In contrast, Dutch adults adhering to the <10 E% added sugar recommendation tended to score lower for the SFA of the healthy diet index indicating a higher SFA intake^(^^26^^)^. In our analysis, butter (source of saturated fat) intake was 28–32 % higher in the highest added sugar intake quartile compared with the lowest quartile, whereas margarine intake was 18–21 % lower and fish group consumption 25–28 % lower in the highest added sugar intake quartile compared with the lowest quartile. These results were not observed with naturally occurring sugar, rather the opposite. Other studies investigating the relationship between sugar types and food consumption indicative of fat quality are few. An American study with 14 709 subjects has, similar to our results, shown a negative relationship between added sugar and fish^(^^14^^)^. Adults adherent to the <10 E% added sugar recommendation were recently found to score higher for the fish component of the Dutch Healthy Diet Index^(^^26^^)^. Overall, further studies are clearly needed to determine the relationship between added sugar intake and dietary fat quality.

We used food groups as crude indicators of diet quality. Studies utilising complementary approaches to disentangle the bearing of added sugar in the diet, such as healthy eating indices or dietary patterns, are still few. The US Department of Agriculture reported that with low EI, high added sugar intake was associated with poorer healthy eating indices compared with those with low intakes of added sugar^(^^33^^)^. One study, using principal component analysis of 7-d dietary records in British adults, revealed that non-milk extrinsic sugar (E%) correlated inversely with the health-conscious dietary pattern^(^^34^^)^. On the other hand, overall diet quality was not associated with adherence to added and free sugar recommendations (<10 E%) in a very recent Dutch study^(^^24^^)^. Hence, further research is needed to fully characterise the relationship between added sugar and dietary quality in the modern, complex food environment. Overall, studies from different countries with divergent food cultures are hard to compare due to divergent methodologies in estimating added sugar intakes^(^^35^^,^^36^^)^.

Our study is subject to limitations. First, our analyses regarding sugar intakes in relation to nutrient intake and food consumption were adjusted for several confounders. Despite this, residual confounding may remain. Second, general difficulties in accurately assessing dietary intakes of free-living individuals apply. This phenomenon reaches far beyond energy-under-reporting, which was taken into account in our analyses. Sugar intake is prone to selective under-reporting, especially among subjects with high BMI^(^^37^^)^. In contrast, fruit and vegetable intakes are likely to be over-reported, thus spuriously increasing the intake of naturally occurring sugar. These viewpoints may explain why added sugar was associated with decreasing waist circumference and BMI in our cross-sectional setting. However, prospective cohort studies, with repeated measurements, are needed to more reliably assess the long-term relationship between added sugar and obesity outcomes. Moreover, the SSB exposure used in most of the studies showing a relationship between added sugar and obesity^(^^1^^,^^3^^)^ should be complemented with other sugar exposures.

Overall, the quantification of the sugar exposures includes inaccuracy. In Finland, sucrose and fructose cover on average 76 and 73 % of total sugars (all mono- and disaccharides excluding lactose) in women and men, respectively. Therefore, the naturally occurring and added sugar terms in our analysis are only approximations of the true intakes and it remains uncertain how the inclusion of the other saccharides (e.g. glucose, galactose and maltose) may have affected the results. Generally, the quality of food composition databases in the coverage of all monosaccharides remains uncertain and calls for efforts in compilation of food composition information. Without this information, reliable monitoring of added sugar intake is unfeasible.

Our added sugar definition is deviant from the free sugar definition used by the WHO^(^^5^^)^. This was, however, reasonable due to the overall moderate consumption of fruit juice in our study population (101 g/d in women and 142 g/d in men). Moreover, the Nordic nutrition recommendations use an added sugar definition similar to our analysis^(^^6^^)^. In addition, increasing added sugar intake was not associated with fruit juice consumption in this study, which suggests that high added sugar consumers not necessarily are high consumers of fruit juice.

Furthermore, a total of 2·9% of the subjects had missing data in one or more components forming the naturally occurring sugar variable. However, excluding these subjects from the main analyses (nutrient and food group association analyses) did not affect the results (data not shown). Impurity of the sugar estimates results also from the calculation procedure. Certain food items (e.g. industrial milk products including real fruits and canned fruits and vegetables, with added sugar) are not disaggregated, and thus some naturally sugar-containing foods may include small amounts of added sugar and vice versa. This was true only for a small proportion of the FFQ food items and the effect on subject misclassification is therefore considered small.

To conclude, this study suggests that naturally occurring and added sugars are associated with divergent food consumption habits, which should be taken into account when studying sugar in relation to health outcomes. A uniform methodology to assess added sugar intake would benefit such efforts and ease cross-study comparisons. The results may inform stakeholders in improving sugar-related communication to foster population health.
